# Biofilm Formation by *Pseudallescheria/Scedosporium* Species: A Comparative Study

**DOI:** 10.3389/fmicb.2017.01568

**Published:** 2017-08-18

**Authors:** Rodrigo Rollin-Pinheiro, Jardel V. de Meirelles, Taissa V. M. Vila, Beatriz B. Fonseca, Vinicius Alves, Susana Frases, Sonia Rozental, Eliana Barreto-Bergter

**Affiliations:** ^1^Laboratório de Química Biológica de Microrganismos, Departamento de Microbiologia Geral, Instituto de Microbiologia Paulo de Goes, Universidade Federal do Rio de Janeiro Rio de Janeiro, Brazil; ^2^Laboratório de Biologia Celular de Fungos, Departamento de Parasitologia e Biologia Celular, Instituto de Biofísica, Universidade Federal do Rio de Janeiro Rio de Janeiro, Brazil; ^3^Laboratório de Ultraestrutura Celular Hertha Meyer, Departamento de Parasitologia e Biologia Celular, Instituto de Biofísica, Universidade Federal do Rio de Janeiro Rio de Janeiro, Brazil

**Keywords:** fungal biofilms, *Scedosporium*, *Pseudallescheria*, virulence, antifungal susceptibility

## Abstract

*Pseudallescheria/Scedosporium* species are medically important fungi that are present in soil and human impacted areas and capable of causing a wide spectrum of diseases in humans. Although little is known about their pathogenesis, their growth process and infection routes are very similar to those of *Aspergillus* species, which grow as biofilms in invasive infections. All nine strains tested here displayed the ability to grow as biofilms *in vitro* and to produce a dense network of interconnected hyphae on both polystyrene and the surfaces of central venous catheters, but with different characteristics. *Scedosporium boydii* and *S. aurantiacum* clinical isolates were able to form biofilms faster than the corresponding environmental strains, as evidenced in kinetic assays for *S. boydii* and CLSM for *S. aurantiacum*. Biofilms formed by *Pseudallescheria/Scedosporium* species had significantly higher resistance to the class of antifungal azole than was observed in planktonic cells, indicating a protective role for this structure. In addition, the clinical *S. aurantiacum* isolate that formed the most robust biofilms was also more virulent in a larvae *Galleria mellonella* infection model, suggesting that the ability to form biofilms enhances virulence in *Pseudallescheria/Scedosporium* species.

## Introduction

*Pseudallescheria/Scedosporium* species are a group of medically important fungi associated with a wide spectrum of infections in both immunocompromised and immunocompetent patients (Cortez et al., 2008). These fungi have a worldwide distribution in soil and human impacted areas, and their disease spectrum ranges from localized skin infections, such as mycetoma, to life-threatening invasive infections, including pulmonary pseudallescheriosis/scedosporiosis, which can potentially disseminate to the central nervous system (Summerbell et al., 1989; Tadros et al., 1998; Guarro et al., 2006; Rougeron et al., 2015). Pseudallescheriosis/scedosporiosis are opportunistic infections that are usually associated with other pathologies, such as cancer, HIV infection, cystic fibrosis and near drowning (Lamaris et al., 2006; Tammer et al., 2011; Zouhair et al., 2013). *Scedosporium (Pseudallescheria) boydii*, *S. apiospermum*, and *S. aurantiacum* are considered clinically relevant species, while others, such as *Pseudallescheria ellipsoidea* and *P. angusta*, are typically classified as environmental species (Guarro et al., 2006; Cortez et al., 2008; Bouchara et al., 2009). Although there has been an alarming increase in *Pseudallescheria/Scedosporium* infections in recent years, little is known about the pathogenesis of these fungi (Al Refai et al., 2002; Lamaris et al., 2006; Wilson and Kennedy, 2013).

Biofilms are complex organized communities that are composed of microbial cells surrounded by a self-secreted extracellular polymeric matrix (Davies, 2003; Kaur and Singh, 2014). Biofilm cells are phenotypically different from their planktonic counterparts and, from a clinical point of view, the most relevant difference is an increased resistance to antimicrobials. The presence of a polymeric extracellular matrix has been shown to confer protection against host immune cells and to impair antifungal penetration (Davies, 2003). *Aspergillus fumigatus* and *Candida albicans* are the most extensively studied pathogenic fungi that cause biofilm-associated invasive fungal diseases (Sherry et al., 2014; Fan et al., 2015). *C. albicans* can adhere to medical devices and form biofilms, and these capabilities have commonly been associated with bloodstream infections (Kojic and Darouiche, 2004). In addition, *Candida* cells that disperse from biofilm structures seem to be more virulent than planktonic cells in animal models (Uppuluri et al., 2010). *A. fumigatus* is able to form biofilms in patients who present with an aspergilloma and invasive aspergillosis (Muller et al., 2011). These infections are established when *A. fumigatus* conidia germinate into mycelia embedded in an extracellular matrix, thereby forming a complex biofilm structure inside the host (Filler and Sheppard, 2006; Kaur and Singh, 2014). Once formed, an *A. fumigatus* biofilm lowers *in vitro* and *in vivo* susceptibility to commercially available antifungal drugs (Seidler et al., 2008; Kaur and Singh, 2014).

Fungi in *Pseudallescheria/Scedosporium* species present a pathogenesis very similar to that of *Aspergillus* species, in which the germination process is crucial for tissue invasion (Bouchara et al., 2009). Nevertheless, the mechanisms underlying the pathogenesis and virulence of *Pseudallescheria/Scedosporium* remain unclear. Recently, it was demonstrated that *S. apios permum*, *S. aurantiacum*, *S. minutisporum*, and *Lomentospora prolificans* can grow as a biofilm on both polystyrene and tissue culture surfaces (Mello et al., 2016). However, no information is available regarding the correlation between the formation of such biofilms and pathogenicity. Hence, in this study, we aimed to compare the ability of environmental and clinical strains of *Pseudallescheria/Scedosporium* species to form biofilms *in vitro* and to correlate these properties with their *in vivo* pathogenicity in a larvae *Galleria mellonella* infection model. Additionally, we sought to evaluate the susceptibility of those biofilms to a variety of antifungal drugs belonging to the classes of azoles and echinocandins.

## Materials and Methods

### Strains and Growth Conditions

The following strains were used in this study: *S. (Pseudallescheria) boydii* CBS 120157, *S. (Pseudallescheria) boydii* CBS 117410, *S. (Pseudallescheria) boydii* CBS 117432, *P. ellipsoidea* CBS 301.79, *P. angusta* CBS 254.72, *S. aurantiacum* CBS 136910, *S. aurantiacum* CBS 136046, *S. aurantiacum* CBS 136047, and *S. aurantiacum* CBS 136049. They were generously provided by Sybren de Hoog from the Westerdijk Fungal Biodiversity Institute, Utrecht, The Netherlands. A list of all nine strains and where they were isolated is shown in **Table 1**. All strains were maintained in modified Sabouraud media (0.5% yeast extract, 1% peptone, and 2% glucose). To obtain conidia, the cells were grown on Sabouraud-agar plates at room temperature, and after 7 days, the surface growth was scraped off using sterile PBS, and the collected conidia were filtered and washed twice with sterile PBS.

**Table 1:** The strains used in this study.

Strains obtained from the CBS collection	Origin	Isolation location	Country
*Scedosporium (Pseudallescheria) boydii* CBS 117410	Environmental	Garden soil	Spain
*Scedosporium (Pseudallescheria) boydii* CBS 117432	Clinical	Sputum of a patient with cystic fibrosis	France
*Scedosporium (Pseudallescheria) boydii* CBS 120157	Clinical	The lung of a patient with leukemia	France
*Pseudallescheria ellipsoidea* CBS 301.79	Environmental	Dung of a cow	Netherland
*Pseudallescheria angusta* CBS 254.72	Environmental	Sewage half of a digestion tank	United States
*Scedosporium aurantiacum* CBS 116910	Clinical	Ankle ulcer	Spain
*Scedosporium aurantiacum* CBS 136046	Clinical	The lung of a patient with an invasive infection	Australia
*Scedosporium aurantiacum* CBS 136047	Environmental	Environmental soil	Australia
*Scedosporium aurantiacum* CBS 136049	Environmental	Soil from a park and playground	Austria

*The nine strains used in this study were obtained from different sources and were generously supplied by the Centraalbureau voor Schimmelcultures (CBS) collection culture*.

### Biofilm Formation

Biofilms were grown on the surface of sterile polystyrene microplates (96- or 24-well), on central venous catheter (CVC) sections or on glass-bottom petri dishes, as previously described (Vila et al., 2015). Briefly, a standardized suspension (10^7^ conidia/ml) was added to each well of the microplate, and the plates were then incubated at 36°C for 1.5 h (adherence phase). After adherence, the supernatant containing the non-adherent cells was removed, and fresh RPMI 1600 media (Sigma-Aldrich, United States) supplemented with 2% glucose and 20% fetal bovine serum (FBS, Gibco, United States) was added to each well. The microplates were then incubated for 30 min or 2, 4, 6, 8, 12, 24, 48, or 72 h. At each time point during incubation, biofilm formation was quantified using two different approaches.

### Biofilm Quantification Assays

Biofilm formation on the surface of polystyrene microplates was quantified using the following two different methods: the overall biomass, which comprised both cells and the ECM, was quantified using crystal violet assays, while the metabolic activity of the cells inside the biofilms, which reflects viability and cell density, was quantified using XTT-reduction assays (Mello et al., 2016).

Crystal violet assay: At each incubation time point, the biofilms were washed with sterile PBS (pH 7.2) to remove any un-adherent cells. The remaining biofilm was fixed in methanol for 15 min and then stained with crystal violet (0.02%) for 20 min. The solution was discarded, and the biofilms were washed two times with PBS. The impregnated crystal violet was dissolved using a 33% acetic acid solution for 5 min, and the colored solution was transferred to a clean microplate and measured using a spectrophotometer at 590 nm (SpectraMAX 340 Tunable; Molecular Devices Ltd., United States).

XTT assay: The biofilms were quantified using a XTT-reduction assay, as previously described (Pierce et al., 2008). First, the biofilms were washed with sterile PBS, and then 100 μl of a XTT: Menadione solution (0.5 mg/ml: 1 μM) was added to the cells. The microplate was incubated for 2 h at 36°C while protected from light. Finally, the colored solution was transferred to a clean microplate, and its absorbance was measured at 490 nm using a spectrophotometer (SpectraMAX 340 Tunable; Molecular Devices Ltd., United States).

### Germination Assay

A standardized suspension containing 10^5^ conidia was added to each well of a microplate with RPMI 1640, and the plates were then incubated at 36°C for 2, 4, 6, and 12 h. At each time point, a total of 200 cells were counted using an optical microscope, and the percentage of germinated cells was calculated.

### Scanning Electron Microscopy (SEM)

The biofilms were grown on the surface of sterile CVC sections (0.5 cm) that were cut longitudinally cut to expose the interior. A catheter section was placed in each well of a 96-well microplate, and biofilms were formed on the inner CVC surface for 24 or 48 h as described above. Then, catheter sections that contained biofilms were processed for scanning electron microscopy (SEM) as previously described (Vila et al., 2013). Briefly, the CVCs were washed in 0.01 M PBS (pH 7.2) and fixed in 2.5% glutaraldehyde and 4% formaldehyde in 0.1 M cacodylate buffer for 1 h at room temperature. Subsequently, the CVCs were washed in the same buffer, post-fixed in 1% osmium tetroxide and 1.25% potassium ferrocyanide for 30 min, and dehydrated in a series of ethanol solutions with increasing concentrations (30, 50, 70, 90, 100% and ‘ultra-dry’ ethanol) for 30 min at each concentration. Then, the samples were critical point-dried in CO_2_, coated with gold and observed using a FEI Quanta 250 scanning electron microscope (FEI, Netherlands).

### Confocal Laser Scanning Microscopy (CLSI)

Biofilms were formed on glass-bottom petri dishes (CellView^TM^, Greiner Bio-One, Germany) as described above. Then, after the biofilms were gently fixed in a 2% formaldehyde solution, they were incubated with the following fluorescent markers: Concanavalin A conjugated to Alexa Fluor 488, Filmtracer^®^Sypro Ruby (both from Molecular Probes, Invitrogen, United States), and Calcofluor White M2R (Sigma-Aldrich, United States). The biofilms were observed using a Leica TCS-SPE confocal scanning microscope (Leica, Germany), and Z-stack reconstructions were analyzed using Fiji software (Schindelin et al., 2012).

### Antifungal Susceptibility Assay

Susceptibility to antifungal drugs was evaluated according to the EUCAST protocols, with some modifications (Taj-Aldeen et al., 2016). Briefly, to each well of a microplate, 100 μl of a standardized suspension (2 × 10^5^ conidia/ml) was added to 100 μl of antifungal drugs serially diluted in RPMI 1640 (supplemented with 2% glucose and buffered with 3-(N-morpholino) propanesulfonic acid (MOPS) 0.165 mol/l, pH 7.0). The concentrations of caspofungin, fluconazole, itraconazole and voriconazole ranged from 0.125 to 128 μg/ml. Control cells were grown in the presence of DMSO (for itraconazole and voriconazole) or water (for caspofungin and fluconazole). The plates were incubated at 36°C for 72 h.

To evaluate biofilm susceptibility, biofilms were formed as previously described (Pierce et al., 2008). Antifungal drugs were added to 24 h-old biofilms at the same concentration range described for planktonic cells. The plates were then incubated for an additional 24 h at 36°C. For both susceptibility tests (planktonic cells and biofilms), a visual reading was performed to define the minimal inhibitory concentration (MIC), and the results were confirmed using a XTT-reduction assay as described above.

### *In Vivo* Assay with *Galleria mellonella*

A survival analysis was performed using the larvae of *G. mellonella* as described by de Lacorte Singulani et al. (2016). Each group of larvae (10 larvae per group) was infected with 10 μl of 10^6^ conidia via the last left pro-leg using a Hamilton syringe. The *S. boydii* CBS 120157, *S. boydii* CBS 117410, *S. aurantiacum* CBS 136046 and *S. aurantiacum* CBS 136047 strains were selected for these experiments. A group of uninfected larvae and a group of uninfected larvae that were inoculated with PBS were used as controls. The larvae were incubated in Petri dishes at 37°C, and population death was evaluated daily for a total of 9 days.

### Statistical Analysis

All statistical analysis were performed using GraphPad Prism 6.0 software (GraphPad, United States). A variance two-way ANOVA was performed using Tukey’s and Bonferroni’s comparisons tests to evaluate the kinetics of biofilm formation in addition to the germination process and survival experiments.

## Results

### Kinetics of Biofilm Formation

**FIGURE 1:**
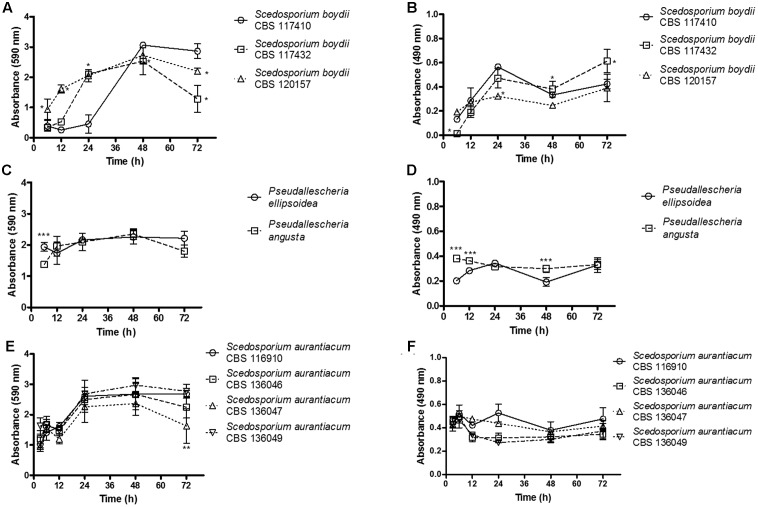
The kinetics of biofilm formation was evaluated using biomass and metabolic assays. Biofilm formation was monitored and quantified using two complementary methods at selected time points (3, 6, 12, 24, 48, and 72 h). The graphs **(A,C,E)** shown demonstrate the total biomass of the biofilms. The results were quantified using a crystal violet assay. The graphs shown in **(B,D,F)** demonstrate the metabolic activity of the cells inside the biofilms. The results were quantified using XTT-reduction assays. Statistical differences (*P* < 0.05) are represented by asterisks. ^∗^Statistical difference when *S. boydii* CBS 117410 was used as a reference. ^∗∗^Statistical difference when *S. aurantiacum* CBS 136049 was used as a reference. ^∗∗∗^Statistical difference when *P. angusta* as was used as a reference.

The growth kinetics of all nine strains that formed biofilms on polystyrene microplates were analyzed by correlating the results of two different analyses: the total biofilm biomass (including cells, both dead and alive, and extracellular matrix), which was evaluated using crystal violet assays (**Figures 1A,C,E**) and metabolic activity, which was quantified using XTT-assays (**Figures 1B,D,F**). These nine strains are representative isolates defined on a meeting of the Working Group on *Pseudallescheria/Scedosporium* Infections, whose researchers received the samples to generate more data about these fungi.

In our analysis of biofilm biomass, among the three *S. boydii* strains, the clinical isolate obtained from human lungs (*S. boydii* CBS 120157) grew significantly faster than the other two isolates (CBS 117432 and CBS 117410), especially at 6 and 12 h (**Figure 1A**). The environmental strain (*S. boydii* CBS 117410) was the slowest-growing strain, and it reached the same biomass as *S. boydii* CBS 120157 only after 48 h. These results suggest that the clinical *S. boydii* strains form biofilms faster than the environmental strain but that all three strains were able to form thick biofilms after 48 h (**Figure 1A**). Interestingly, the *S. boydii* clinical strain (CBS 120157) had significantly lower metabolic activity inside the biofilm at 24 h, but at 48 h, all three *S. boydii* strains had similar metabolic activity (**Figure 1B**). The typical environmental species *P. ellipsoidea* and *P. angusta* produced a similar amount of biomass and *P. angusta* exhibited a significantly higher metabolic rate at 6, 12, and 48 h (**Figures 1C,D**).

The *S. aurantiacum* clinical isolates shared a similar pattern of biomass growth, and both strains produced biomass levels that were the same as that produced by the *S. boydii* clinical isolates within 24 h (**Figure 1E**). Remarkably, across all of the *S. aurantiacum* strains, there was no significant difference in the amount of biomass produced or metabolic activity rates, excepting at 72 h when *S. aurantiacum* CBS 136047 presented significantly less biomass comparing to the other strains (**Figures 1E,F**).

### Conidial Germination Assay

**FIGURE 2:**
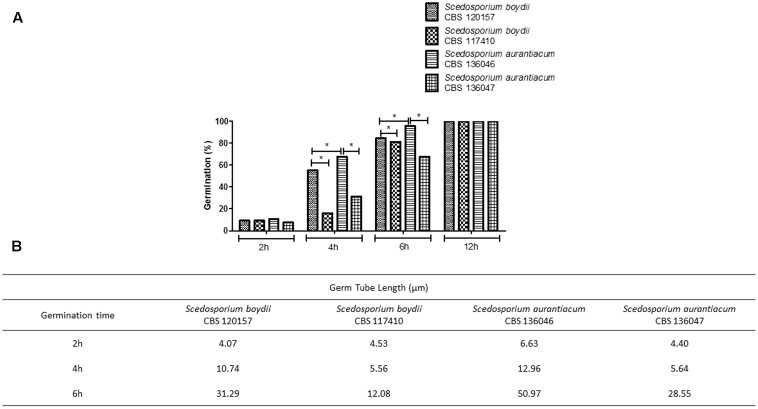
Germ tube formation in clinical and environmental strains of *Scedosporium boydii* and *S. aurantiacum*. **(A)** The germination process was monitored over time and quantified by counting the percentage of germinated cells at selected time points (2, 4, 6, and 12 h). **(B)** Germ tube length was monitored during the germination process at selected time points (2, 4, or 6 h). Statistical differences (*P* < 0.05) are represented by asterisks.

For the further analysis, two strains of *S. aurantiacum* and *S. boydii* were chosen, since the experiments present a significantly high cost to be performed with all nine isolates. A germination assay was performed using fresh conidia. One clinical and one environmental strain of each species (*S. boydii* CBS 120157 and CBS 117410 and *S. aurantiacum* CBS 136046 and CBS 136047) were selected to evaluate growth profiles (**Figure 2**). For all of the included strains, regardless of the germ tube length, approximately 10% of the cells were germinating after 2 h of incubation, and 100% were germinating after 12 h (**Figure 2A**). The most important difference between the germination profiles was observed after 4 and 6 h of incubation when the clinical isolates, *S. boydii* CBS 120157 and *S. aurantiacum* CBS 136046, were found to have germinated 2- to 3-fold faster than the environmental isolates, *S. boydii* CBS 117410 and *S. aurantiacum* CBS 136047 (**Figure 2A**). Similar results were observed when the germ tube lengths were measured, as follows: although similar germ tube lengths were observed after 2 h of incubation, the tube lengths were different after 4 h of incubation (**Figure 2B**).

### Confocal Scanning Microscopy of *Scedosporium/Pseudallesheria* Biofilms

To compare the biofilm structures of clinical and environmental strains of *S. boydii* and *S. aurantiacum*, we selected one clinical and one environmental strain of each species (*S. boydii* 120157 and 117410, respectively, and *S. aurantiacum* 136046 and 136047, respectively) and imaged the biofilms using CLSM. Biofilm cells were stained with (a) Concanavalin A, which stains glucose and mannose residues, both of which are present in cell walls and biofilm ECM; (b) Calcofluor white, which stains chitin structures in the cell walls of fungi, and (c) Filmtracer^®^sypro ruby, which stains glycoproteins known to be abundant in the ECM of other fungi.

**FIGURE 3:**
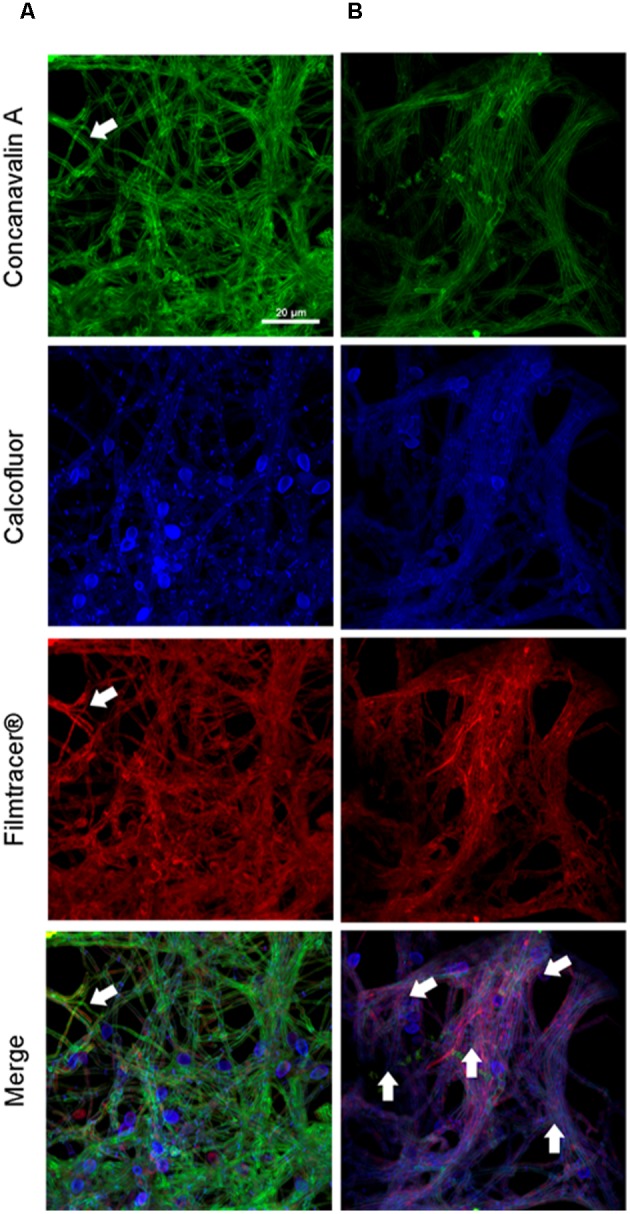
Confocal laser scanning microscopy of *S. boydii* biofilms grown for 12 h *in vitro*. **(A)** *S. boydii* CBS 120157 and **(B)** *S. boydii* CBS 117410. In the panels, glucose-mannose residues were stained using concanavalin A-Alexa 488 (green panel), cell walls and matrix chitin were stained using calcofluor white (blue panels) and glycoproteins were stained using the biofilm-tracer FilmTracer^®^Sypro ruby (red panels). White arrows indicate accumulated extracellular matrix. The same magnification (63×) was used in all panels.

**FIGURE 4:**
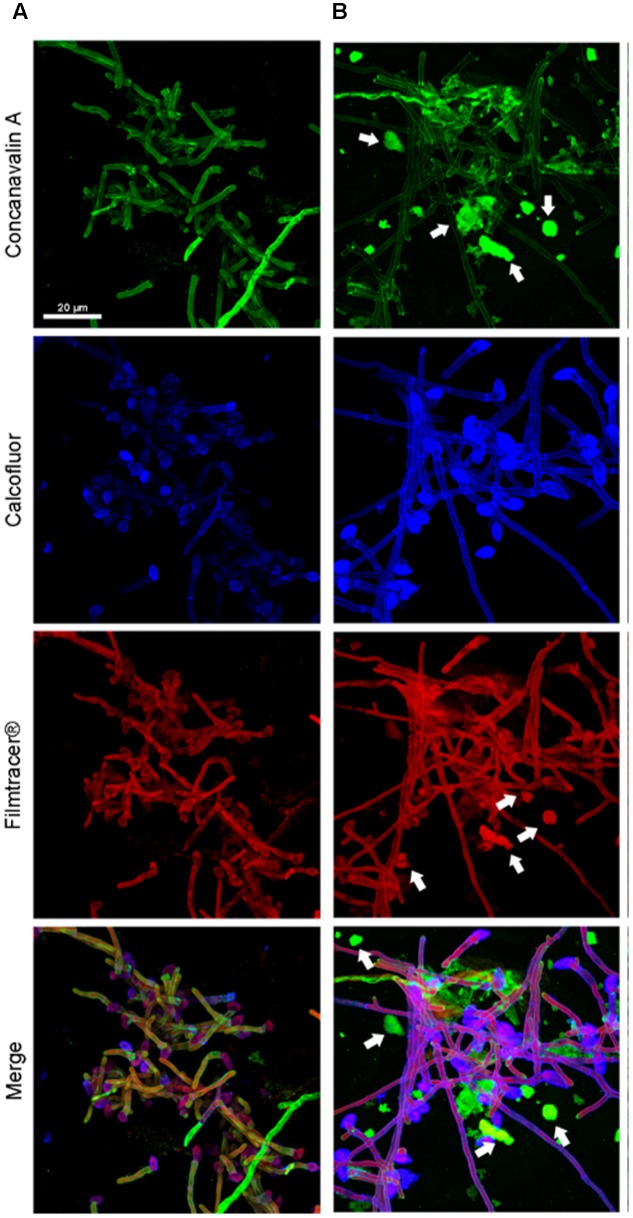
Confocal microscopy of a *S. aurantiacum* biofilms grown for 6 h *in vitro*. **(A)** *S. aurantiacum* CBS 136046 and **(B)** *S. aurantiacum* CBS 136047. In the panels, glucose-mannose residues were stained using concanavalin A-Alexa 488 (green panel), cell walls and matrix chitin were stained using calcofluor white (blue panels) and glycoproteins were stained using the biofilm-tracer FilmTracer^®^Sypro ruby (red panels). White arrows indicate accumulated extracellular matrix. The same magnification (63×) was used in all panels.

A more robust biofilm was formed by the *S. boydii* clinical isolate (120157) than the corresponding environmental strain (117410) (**Figures 3A,B**). These results corroborate the data shown in **Figures 1**, **2**. However, there was little to no visible difference between the biofilms formed by the clinical and the environmental strains of S. *aurantiacum* (**Figures 4A,B**).

### Scanning Electron Microscopy of *Scedosporium/Pseudallesheria* Biofilms

Biofilms were formed on catheters using the clinical isolates of each species (i.e., *S. boydii* CBS 120157 and *S. aurantiacum* CBS 136046) with the aim of evaluating their ability to adhere to and develop biofilms on medical devices. The biofilms were visualized using SEM.

**FIGURE 5:**
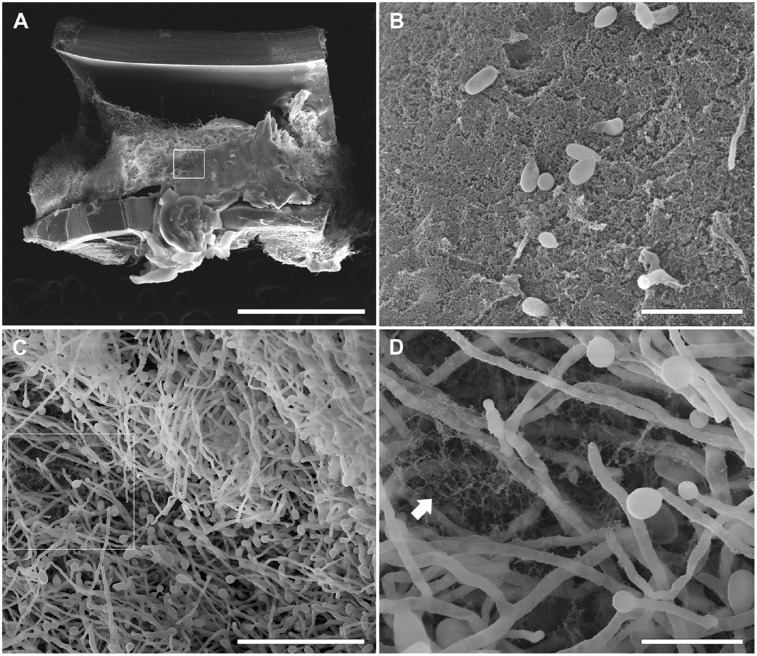
Scanning electron microscopy of *S. boydii* clinical isolate (CBS 120157) biofilms grown on central venous catheters (CVCs). Biofilms were grown *in vitro* on the inner surface of 0.5 cm sections of sterile CVCs for 48 h. **(A)** A dense mass of *S. boydii* biofilm covered most of the CVC surface. **(B)** The inset of the white square shown in panel **(A)** shows an area of dense extracellular matrix (ECM; white arrow) and a dispersion of conidia. **(C)** A representative area of the internal biofilm. **(D)** An inset of the white square in panel. **(C)** showing a network of hyphae with ECM residues. Bars correspond to 1 mm **(A)**, 20 μM **(B)**, 50 μM **(C)**, and 10 μM **(D)**.

**FIGURE 6:**
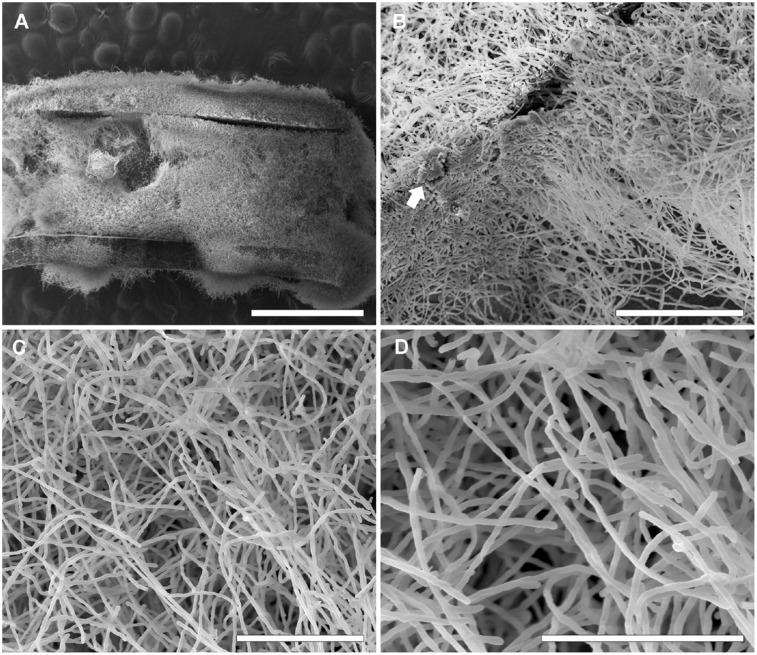
Scanning electron microscopy of *S. aurantiacum* clinical isolate (CBS 136046) biofilms grown on CVCs. Biofilms were grown *in vitro* on the inner surface of 0.5 cm sections of sterile CVCs for 24 h. **(A)** A dense mass of *S. aurantiacum* biofilms covered the entire CVC surface. **(B,C)** A representative area of an internal area of a biofilm with a dense network of hyphae (white arrow). **(D)** A higher magnification of the central area shown in panel **(C)**. The bars correspond to 1 mm **(A)**, 100 μM **(B)**, 50 μM **(C)**, and 40 μM **(D)**.

Similar to the results previously described for the biofilms grown on polystyrene and glass-bottom surfaces in this study, biofilms formed faster on CVCs in the *S. aurantiacum* clinical isolate than in the *S. boydii* clinical isolate. Whereas 24 h was sufficient for *S. aurantiacum* to colonize the entire catheter surface (**Figure 6**), the *S. boydii* isolate required 48 h to reach a similar biomass density (**Figure 5**). The presence of ECM was observed among hyphae that had adhered to the catheter surface, especially in *S. boydii* and less in *S. aurantiacum*, suggesting the formation of a mature biofilm structure (arrows in **Figures 5D**, **6B**).

### Biofilm Formation on Different Abiotic Surfaces

**FIGURE 7:**
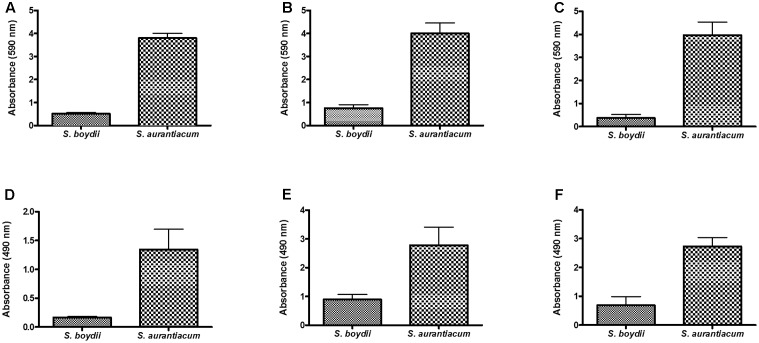
Comparison of biofilm formation by *S. boydii* and *S. aurantiacum* on three different surfaces. Biofilm formation was evaluated after 24 h of growth. The total biomass of the biofilms was quantified using a crystal violet assay **(A,B,C)**, and the metabolic activity of the cells inside the biofilms was quantified using a XTT-reduction assay **(D,E,F)**. Three different surfaces were used, including glass Petridish bottoms **(A,D)**, polystyrene **(B,E)**, and catheters **(C,F)**. Statistical differences (*P* < 0.05) are represented by asterisks.

To confirm that *S. boydii* and *S. aurantiacum* grow in a similar manner on all of the different surfaces used in this study (i.e., polystyrene, glass bottom dishes and catheters), biofilm formation was simultaneously measured using crystal violet and XTT-reduction assays in clinical isolates of *S. boydii* (CBS 120157) and *S. aurantiacum* (CBS 136046) that were grown on all three surfaces. After 24 h, both isolates had adhered to and grown biofilms on all three surfaces and, as expected, faster growth was observed on all 3 surfaces by *S. aurantiacum* as compared with *S. boydii* (**Figure 7**).

### Antifungal Susceptibility of Biofilms and Planktonic Cells

Because increased resistance to drugs is the most clinically relevant phenotypical alteration observed in biofilms, we evaluated the susceptibility of *S. boydii* and *S. aurantiacum* strains to different antifungal drugs and compared the results to those obtained for their planktonic counterparts (primary suspension inoculums were used to form the biofilms). As expected, the biofilms were generally less susceptible to all the azole drugs (MIC > 128 μg/ml) than were their planktonic cells. Interestingly, caspofungin was the only drug for which we obtained a lower MIC value in the biofilms (32 μg/ml) than in the planktonic cells (64 μg/ml) (**Table 2**).

**Table 2:** The minimum inhibitory concentration (MIC) of different antifungal drugs used to treat *S. boydii* and *S. aurantiacum* planktonic cells and biofilms.

Fungi	Antifungal drugs (range 128–0.125 μg/ml)	MIC of planktonic cells (μg/ml)	MIC of biofilms (μg/ml)
*Scedosporium (Pseudallescheria)*	Caspofungin	64	32
*boydii* CBS 120157	Fluconazole	32	>128
	Itraconazole	2	>128
	Voriconazole	1	>128
*Scedosporium (Pseudallescheria)*	Caspofungin	64	32
*boydii* CBS 117410	Fluconazole	64	>128
	Itraconazole	2	>128
	Voriconazole	0.5	>128
*Scedosporium aurantiacum* CBS	Caspofungin	64	32
136046	Fluconazole	16	>128
	Itraconazole	1	>128
	Voriconazole	0.5	>128
*Scedosporium aurantiacum* CBS	Caspofungin	64	32
136047	Fluconazole	32	>128
	Itraconazole	1	>128
	Voriconazole	0.5	>128

*An antifungal susceptibility test was performed using planktonic cells and pre-formed biofilms*.

In the planktonic cells, itraconazole (MIC, 1–2 μg/ml) and voriconazole (MIC, 0.5–1 μg/ml) were the most active antifungals. As was expected for filamentous fungi, these isolates were less sensitive to fluconazole (MIC between 16 and 64 μg/ml) (**Table 2**). No relevant difference was observed in antifungal susceptibility between these distinct genera and or by sample source.

### *In Vivo* Virulence Assay Using *Galleria mellonella*

**FIGURE 8:**
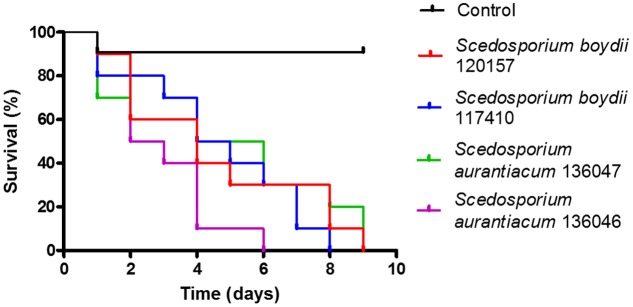
*In vivo* infection model of *Galleria mellonella*. An *in vivo* infection model was performed in which we infected 10 larvae per group with 10^6^ conidia of one of the strains used in this study. The control group was inoculated with PBS. Each population was observed daily for 9 days. The survival results are shown. Statistical differences (*P* < 0.05) are represented by asterisks.

An *in vivo* infection model was established using larvae of *G. mellonella*. In this assay, we evaluated the pathogenicity of the strains used in this study to construct survival curves (**Figure 8**). Interestingly, the *S. aurantiacum* clinical isolate (136046), which had the fastest germ tube elongation (**Figure 2B**), was also the most virulent strain in that it had caused 100% mortality in *G. mellonella* at 6 days after infection (**Figure 8**). The other three strains (*S. aurantiacum* 136047, *P. boydii* 120157 and *S. boydii* 117410) had similar virulence patterns and had killed the larvae by 8–9 days post-infection (**Figure 8**).

## Discussion

The ability of some *Pseudallescheria/Scedosporium* species, such as *S. apiospermum*, *S. aurantiacum*, *S. minutisporum*, and *L. prolificans*, to form biofilms was recently described by Mello et al. (2016). In this study, Mello et al. reported that both species can adhere to polystyrene surfaces and lung epithelial cells, on which they form biofilms that are less susceptible to antifungal drugs. Additionally, the authors characterized biofilm organization, and their results indicated that the clinically relevant *Pseudallescheria*/*Scedosporium* species formed biofilms. In the present study, we compared the ability of clinical and environmental isolates of two *Scedosporium* species to germinate and grow as biofilms *in vitro* on different surfaces. The correlation between biofilm formation and antifungal susceptibility or virulence was also examined.

Our results show that biofilm formation is faster in *S. aurantiacum* than in *S. boydii* (**Figures 1**, **7**), as previously described by Mello et al. (2016). Interestingly, the metabolic activity of the *S. boydii* biofilms was higher than that of the *S. aurantiacum* strains (**Figure 1**). Because the metabolic activity of the biofilms formed by all of the tested strains was not altered during the course of the kinetics assay, and because our visual observations showed that there was an increase in fungal growth, we hypothesized that the abundant secretion of ECM in biofilms could impair XTT penetration, resulting in a reduced color change reaction that may have compromised the accuracy of the assay.

After 12 h, the *S. boydii* clinical isolate (CBS 120157) had formed thicker biofilms than was observed in the environmental strain (CBS 117410), as demonstrated by the kinetics assay and CLSM (**Figures 1**, **3**). Interestingly, according to CLSM, after 6 h the clinical isolate of *S. aurantiacum* (CBS 136046) had formed less biofilm than was observed in the environmental isolate (CBS 136047) (**Figure 4**). However, the presence of ECM among the interconnected hyphae was more evident in the CLSM images of the clinical isolate (CBS 136046) (**Figure 4**). In *S. boydii* (CBS 120157), ECM could be well observed in SEM pictures (**Figure 5**). In the biofilms of both species, ECM glycoproteins were homogeneously distributed around the mycelial mass and some glucose-rich residues. The ECM is a crucial structure for robust biofilms that contributes to the increase in resistance observed in these communities (Borghi et al., 2016). Therefore, determining the composition of the ECM is very important for understanding and predicting drug-resistance in biofilms. The ECM composition varies among different fungal species and even across different environmental conditions under which biofilm can grow (Al-Fattani and Douglas, 2006; Flemming and Wingender, 2010; Reichhardt et al., 2015). As far as we are aware, the ECM composition of *Pseudallescheria* and *Scedosporium* biofilms is unknown, and further studies are therefore needed to determine its composition and whether it plays an important role in drug-sequestration, as was described in *Candida* biofilms (Nett et al., 2010; Vediyappan et al., 2010; Mitchell et al., 2016).

Biofilm formation is an important risk factor for mortality in patients with bloodstream infections of *C. albicans*(Rajendran et al., 2016), especially because these biofilms can grow on the surface of any inserted devices and thereby create a continuous cycle of reinfection. Reports showing that *S. boydii* and *S. apiospermum* caused catheter-related infections have been published (Perez et al., 1988; Eldin et al., 2012). Here, we used SEM to demonstrate that *S. boydii* and *S. aurantiacum* clinical isolates obtained from lung infections can form robust biofilms *in vitro* on CVC surfaces (**Figures 5**, **6**). SEM images showed that a multicellular biofilm had formed and that it was composed of a dense network of hyphae on which several conidia were ready to be released from the end of mycelia. In addition, the presence of ECM among the mycelia was also observed, and these data corroborated those found in the CLSM analysis. The biofilms formed by *S. boydii* and *S. aurantiacum* inside the CVCs were very similar to those previously reported for *Aspergillus* sp. (Kaur and Singh, 2014) and other *Pseudallescheria/Scedosporium* species (Mello et al., 2016). These data show that *Pseudallescheria/Scedosporium* species are capable of forming biofilms on non-biotic surfaces, which reinforces their potential to cause opportunistic infections in healthcare units.

Fungal germination is the initial step in mycelia and biofilm formation and, therefore, the germination rate of each species might be important for predicting its biofilm formation profile. As expected, both clinical isolates germinated faster than their environmental counterparts, and the *S. aurantiacum* clinical isolate was the fastest strain to germinate. To our knowledge, this is the first study to compare biofilm formation and germination using different clinical and environmental isolates obtained from the *Pseudallescheria/Scedosporium* species. Similar results were previously demonstrated by Nunes et al. (2013) in *Rhodotorula mucilaginosa*, an emerging yeast pathogen that causes invasive infections and in which the clinical isolates were more capable of forming biofilms than were their environmental isolates (Nunes et al., 2013). In *Candida* spp., the formation of biofilms by clinical isolates was associated with higher mortality in patients with candidemia and was considered a significant predictor of mortality in hospitalized patients (Tumbarello et al., 2007; Rajendran et al., 2016). Conversely, an *S. aurantiacum* clinical isolate was found to be more virulent *in vivo* than an environmental strain of this species (**Figure 8**). In fact, this isolate had the highest virulence of all strains tested and had killed 90% of the population in 4 days. The clinical isolate of *S. aurantiacum* also produced a more substantial biofilm than was produced by the clinical isolate of *S. boydii* and was the fastest strain to germinate, suggesting that there is a correlation between the speed of germination, biofilm formation and virulence.

Biofilms are structures that have well-known associations with increased levels of antifungal resistance (Borghi et al., 2015). Here, we show that *Scedosporium* biofilms are less susceptible to all azoles than are their planktonic counterparts (**Table 2**). The role of biofilms in antifungal resistance has been extensively studied in *Aspergillus* and *Candida.* It is widely accepted that the glucan-enriched ECM of *C. albicans* biofilms sequesters antifungal drugs and reduces susceptibility to them (Nett et al., 2010). Additionally, efflux pumps have been identified in both *C. albicans* and *Aspergillus* biofilms, in which they lead to resistance to azoles (Rajendran et al., 2011; Ramage et al., 2011). Hence, further studies are needed to identify the mechanisms involved in the increased resistance of *Pseudallescheria/Scedosporium* biofilms.

In summary, our results demonstrate that clinical and environmental strains of *Pseudallescheria/Scedosporium* form biofilms on different abiotic surfaces and that this process is correlated with antifungal susceptibility and virulence *in vivo*. Because *Pseudallescheria/Scedosporium* species are pathogens that are increasing in frequency worldwide and because most such infections are resistant to the available antifungal arsenal, obtaining a better understanding of the role of biofilms in hospital-acquired cases of pseudallescheriosis and scedosporiosis is increasingly important. However, determining how biofilms contribute to their pathogenesis would allow us to better evaluate the effectiveness of treatments for these infections.

## Author Contributions

RR-P, JdM, TV, BF, and VA conceived, designed and performed the experiments. RR-P, JdM, TV, SF, SR, and EB-B analyzed the experiments. RR-P, JdM, TV, SR, and EB-B drafted the manuscript.

## Conflict of Interest Statement

The authors declare that the research was conducted in the absence of any commercial or financial relationships that could be construed as a potential conflict of interest.
